# Reliability and Validity Measurement of Sagittal Lumbosacral Quiet Standing Posture with a Smartphone Application in a Mixed Population of 183 College Students and Personnel

**DOI:** 10.1155/2016/3817270

**Published:** 2016-10-23

**Authors:** George A. Koumantakis, Maria Nikoloudaki, Sara Thacheth, Kalliroi Zagli, Konstantina Bitrou, Andreas Nigritinos, Leon Botton

**Affiliations:** ^1^401 General Army Hospital of Athens, Pan. Kanellopoulou 1, Athens, Greece; ^2^Athens Metropolitan College, Health Sciences Faculty, School of Physiotherapy, Athens, Greece

## Abstract

Accurate recording of spinal posture with simple and accessible measurement devices in clinical practice may lead to spinal loading optimization in occupations related to prolonged sitting and standing postures. Therefore, the purpose of this study was to establish the level of reliability of sagittal lumbosacral posture in quiet standing and the validity of the method in differentiating between male and female subjects, establishing in parallel a normative database. 183 participants (83 males and 100 females), with no current low back or pelvic pain, were assessed using the “iHandy Level” smartphone application. Intrarater reliability (3 same-day sequential measurements) was high for both the lumbar curve (ICC_2,1_: 0.96, SEM: 2.13°, and MDC_95%_: 5.9°) and the sacral slope (ICC_2,1_: 0.97, SEM: 1.61°, and MDC_95%_: 4.46°) sagittal alignment. Data analysis for each gender separately confirmed equally high reliability for both male and female participants. Correlation between lumbar curve and sacral slope was high (Pearson's *r* = 0.86, *p* < 0.001). Between-gender comparisons confirmed the validity of the method to differentiate between male and female lumbar curve and sacral slope angles, with females generally demonstrating greater lumbosacral values (*p* < 0.001). The “iHandy Level” application is a reliable and valid tool in the measurement of lumbosacral quiet standing spinal posture in the sagittal plane.

## 1. Introduction

Sagittal lumbopelvic alignment is an important physical assessment parameter for the orthopaedic [1] and physical therapy [2] health professionals. Posture is defined as the alignment and positioning of the body in relation to gravity, center of mass, or base of support in order to minimize load [3] and prevention against potential injury [2]. The sagittal curvatures of the spine [4] and the sagittal alignment of the pelvis [5] represent the basic mechanism for maintaining an efficient standing position [6].

Postural deviations from the “neutral” range [7] are considered to be a significant reason for the appearance of back pain, according to several studies [8–12]. Different methods to assess sagittal spinal posture have been used, in the light of poor reliability demonstrated with visual assessment of posture [13], with X-rays on one hand being the gold-standard assessment method [4], however not routinely used for postural evaluation due to associated costs and subject irradiation. Vrtovec et al. [4] in an excellent review analysed all potential X-ray based methods of posture analysis. Indeed, the accuracy of radiographic methods of postural assessment is high, with interrater Intraclass Correlation Coefficient (ICC) and Minimum Detectable Change (MDC) values of 0.98 and 3.9°, respectively, for lumbar curve assessments in a relevant study [14]. Photographic methods have also been used comparatively to X-ray methods, with acceptable correlations between the 2 methods in determining the size of the Cobb angle and the length of lordosis; however, values with the photogrammetric method were systematically lower compared to X-rays [15].

For screening purposes of lumbosacral posture in large populations, several more easily applied, low-cost, accurate, and valid assessment methods, such as body contour and inclinometric posture measurements, have been developed. Youdas et al. (1996) in a reliability study of 90 healthy participants, using the flexible curve method (tangential calculation of angles) for the lumbar curve and an inclinometer for the pelvic inclination (sacral slope), reported intrarater ICC_1,1_ reliability values of 0.82 for lumbar curve and 0.91 for sacral slope measurements. Mean ± standard deviation (SD) values for lumbar curve measured in standing were 37.5 (11.0)° for men and 52.7 (15.3)° for women and for sacral slope were 13.8 (4.5)° for men and 22.8 (7.6)° for women [16]. Another study comparing the reliability of 2 different noninvasive methods of lumbar curve estimation performed in 30 participants reported intrarater ICC_3,3_ values of 0.90–0.92 with the Metrecom computer-interfaced digitizer (tangential and trigonometric calculation of angles) and of 0.92 for the inclinometric (tangential calculation of angles) method [17]. A study using the Saunders digital inclinometer for sagittal posture measurement in standing in 30 healthy participants had adequate intrarater reliability, with measurement error values of 3.2° for lumbar curve and 3.3° for sacral slope [18]. Mean (SD) values of 2 measurements taken a week apart from the same examiner for lumbar curve were 32.7–34.3 (7.5–8.0)° and for sacral slope 19.6–20.6 (5.9)° [18].

Lately, digital inclinometers in the form of smartphone applications have emerged as alternative measurement methods [19]; however, thorough validation of smartphones for goniometric measurements is required before their extensive use. A recent study [20] accurately recorded the intra- and interrater reliability of the lumbar curve sagittal standing posture in 30 healthy participants; intrarater reliability values of this study are reported in Table 2(b). Also, this study reported equally high interrater ICC_2,*k*_ reliability values of 0.96 for smartphone measurements and of 0.90 for bubble inclinometer measurements [20].

The purpose of our study was to examine the intrarater reliability of the method in a sample larger than previously reported [20], to identify whether posture measurements are affected by gender, to assess the correlation between pelvic posture and lumbar posture, and to establish a normative database for future reference.

## 2. Methods

### 2.1. Participants

One hundred and eighty-three healthy adults (83 males and 100 females) volunteered to participate. All participants were students or employees from various faculties at Akmi Metropolitan College and were verbally invited to participate to the research by team members. The mean (SD) age, height, body mass, and BMI of participants were 26.1 (10.04) years, 172 (9.4) cm, 68.7 (14.5) kg, and 22.8 (3.6) kg/m^2^, respectively. Exclusion criteria were to have active low back pain and/or trauma and for female participants' menstruation, in order to ensure the confounding effects of current pain presence in body posture of the participants included in the investigation. All participants included in the study were approached via notice-board and e-mail announcements. They all signed a written informed consent, presenting the exclusion criteria and the aims and purposes of the study prior to their participation. The study protocol was approved by the Ethics Committee of the Athens Metropolitan College. All rights of participants were protected at all times, according to the declaration of Helsinki.

### 2.2. Instrumentation

Smartphones with the Android operating system were used, with the “iHandy Level” application installed. The “iHandy Level” application is a tool that has already been validated for the measurement of spinal range of movement [21] and posture evaluation [20]. It is a free application, which has a visual display alike with the digital inclinometer in regard to numeric size. Salamh and Kolber (2014) [20] state that the application uses the smartphones' Android built-in accelerometer and a digital display to show the angle measured.

### 2.3. Pilot Study and Research Design

The pilot study was conducted between the members of the team (10 participants), to accustom themselves with the measurement procedure. Participants were asked to stand in a comfortable position with their arms at their side and their lower limbs parallel to each other. Three sequential measurements of pelvic and low back posture were performed, to establish the reliability of the procedure. Between measurements, the participants were asked to perform 10 steps, to alter their posture between the measurements.

Each team member was assigned a task prior to the commencement of measurements: one member was responsible for verbally informing the participants of the purposes of this research and the measurement procedures involved and for collecting the participants demographic details, two members were involved in the palpation and skin-marking, two were involved in the measurement process, and one member recorded the posture data obtained for the two members involved in the actual measurement procedure to be blinded to the recording of results.

### 2.4. Measurement Procedure

Participants stood with their low back area from T12-top of sacrum exposed for the skin-marking and palpation procedures. Our measurement method took into account that the sacral slope (SS) corresponds to the angle between the upper sacral endplate and the horizontal plane [5] and that the lumbar curve is defined as the angle between the upper endplates of L1 and S1 [22]. Therefore, for spine and pelvis accurate marking, the researchers had to locate the 12th rib and palpate it towards the spine in order to locate the 12th thoracic vertebra. They also palpated the iliac crests, to locate the interspace between the spinous processes of the 4th and 5th lumbar vertebra (L4-L5). Subsequently the spinous processes of the 1st and 2nd sacral vertebra (S1-S2) were identified one and two levels below L5. Skin-marking was made with a dry-erase marker at T12-L1 and S1-S2 spinous processes.

**Figure 1:**
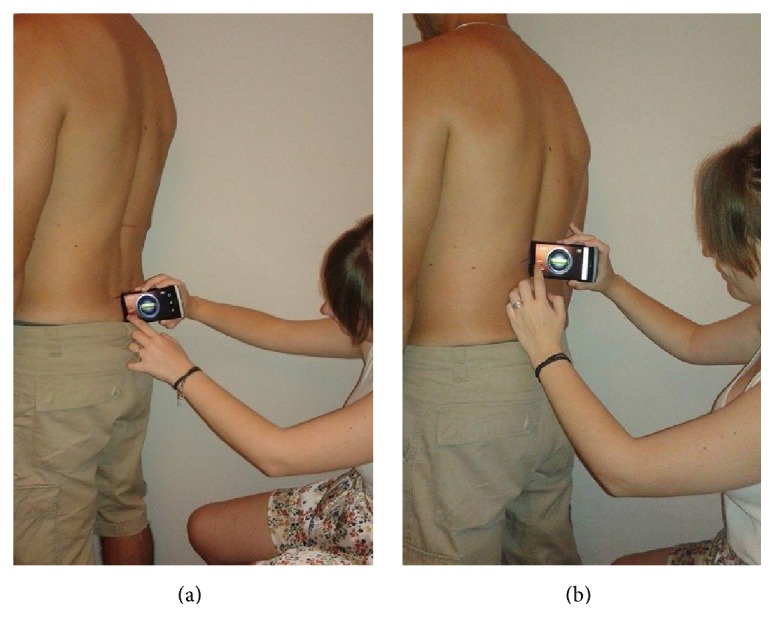
Sacral slope and lumbar curve measurement technique with the smartphone placed at (a) S1-S2 interspace and (b) T12-L1 interspace.

The last step of the investigation was to conduct the measurement with the “iHandy Level” application tool. The researchers calibrated (zeroed) the application's indication on a stable level surface before obtaining the 3 sequential measurements. This calibration procedure was followed for each participant separately. The angle readings from the smartphone, when placed with its upper vertical side at T12-L1 and S1-S2 spinous processes, were recorded (Figure 1). The sacral slope value corresponded to the reading from S1-S2 and the lumbar curve value corresponded to the sum of the absolute readings from T12-L1 and S1-S2. Participants altered their posture in between the measurements by taking 10 steps before reassuming a relaxed standing posture in order to be remeasured. The aforementioned procedure was conducted three times.

The methodology followed in our study is the double inclinometer method, followed by Waddell et al. 1992, used to assess spinal movement, by placing an electronic goniometer at T12-L1 and S1-S2 landmarks in upright standing and at end-range spinal movements [23]. It is also similar to the tangential angle calculation method used in studies with the flexible curve ruler [16, 24, 25], whereby the curvature is calculated by the angle formed by 2 tangents drawn at the end-points of the curve. Other studies have shown there can be a marked difference between the tangential and the trigonometric method of calculation of spinal curves [17]; however, the tangential method has been shown to correlate with angle analysis through X-rays [24].

### 2.5. Data Analysis

All statistical analyses were performed using the Statistical Package for Social Sciences (SPSS), version 20. Descriptive data including mean measurement angles with standard deviations were calculated for each series of measurements. All data were tested with the Kolmogorov-Smirnov test, and it was verified that they were normally distributed.

Repeated measures ANOVAs were performed between the 3 measurements of sacral slope and lumbar curve separately and the level of significance was set at *p* = 0.05, to identify possible significant systematic trends between the 3 measurement occasions [26]. The reliability of all measurements was determined by the ICC Model 2,1 absolute agreement for the intrarater component of analysis. According to Portney and Watkins (2014) [26], a value of above 0.75 in the ICC value is classified as good. The repeatability and the precision of the measurement were additionally described using the Standard Error of the Measurement ($SEM=SD\sqrt{1-ICC}$) [26]. The Minimum Detectable Change (MDC) was calculated for the intrarater measurements using the formula MDC_95%  CI_ = SEM × 1.96 × $\sqrt{2}$, to determine the magnitude of change which will exceed the threshold of measurement error at the 95% confidence level [26]. Finally, graphical representations of agreement between the 3 measurements were depicted through mean differences and 95% limits of agreement (LOA) with Bland and Altman plots [27], comparing measurements 1 and 2, 2 and 3, and 1 and 3 in 3 separate plots per angle measured.

Construct validity represents a postulated attribute of participants like gender and its associated characteristics or relationships between attributes that are assumed to be reflected in test performance [26]. As measures of construct validity of the methodology of postural measurement tested, correlations between the 2 angles (lumbar curve and sacral slope) were made for the whole group and for male and female participants separately, using Pearson's correlation coefficient to determine the level of relationships between the variables examined. Also, the differences between genders were examined for both lumbar curves and sacral slope with independent samples of *t*-test.

## 3. Results

### 3.1. Demographics

According to the descriptive categorical statistics, 54.6% of the participants were females and 45.4% were males. Most of the participants were students 77.6% (142/183) and a smaller percentage 22.4% (41/183) were administrative personnel.

### 3.2. Lumbosacral Angle Data

Data for the 3 testing occasions for both lumbar curves and sacral slopes are analytically presented in Table 1. Data of the same angles (mean of 3 measurements) are separately presented for male and female participants (Table 2(a)).

### 3.3. Reliability and Agreement

Repeated measures of ANOVA analysis for both angles were almost at or above *p* = 0.05. The value of *p* = 0.047 for sacral slope is very close to the cut-off significance level and was accepted as almost nonsignificant, although this reflects a difference between measurement 1 and measurements 2 and 3 which were closer together. This pattern is also reflected in the Bland and Altman plots (Figure 3).

Based on the results shown in Table 2(b), the reliability of “iHandy Level” application for both lumbar curve and sacral slope measurements was high for the overall group and also for male and female participants examined separately, with all ICC values estimated between 0.93–0.97. Similarly, SEM and MDC values were relatively low. SEM values were between 2.02° and 2.23° for lumbar curve and even lower between 1.57° and 1.63° for sacral slope angles. The MDC (95% CI) values, above which the change in measurement can be considered as due to true change and not due to repeated measurement of the same state, were 5.9° for lumbar curve and 4.46° for sacral slope.

**Figure 2:**
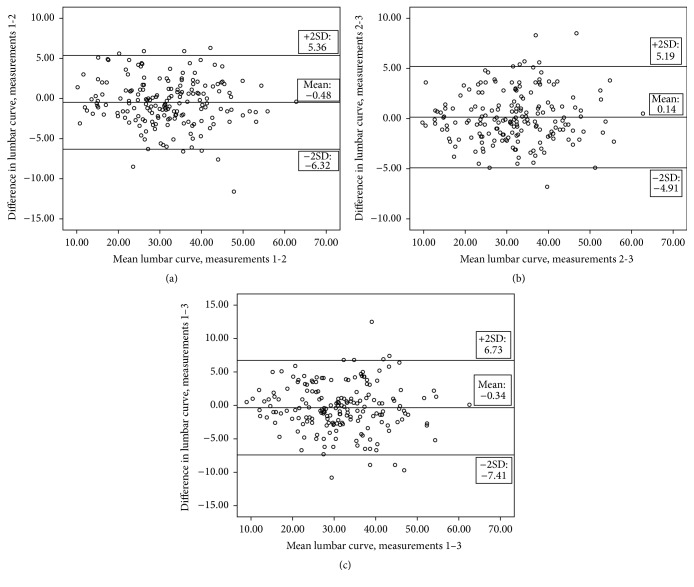
Bland and Altman plots for lumbar curve measurements, indicating mean differences and 95% limits of agreement between (a) measurements 1 and 2, (b) measurements 2 and 3, and (c) measurements 1 and 3.

**Figure 3:**
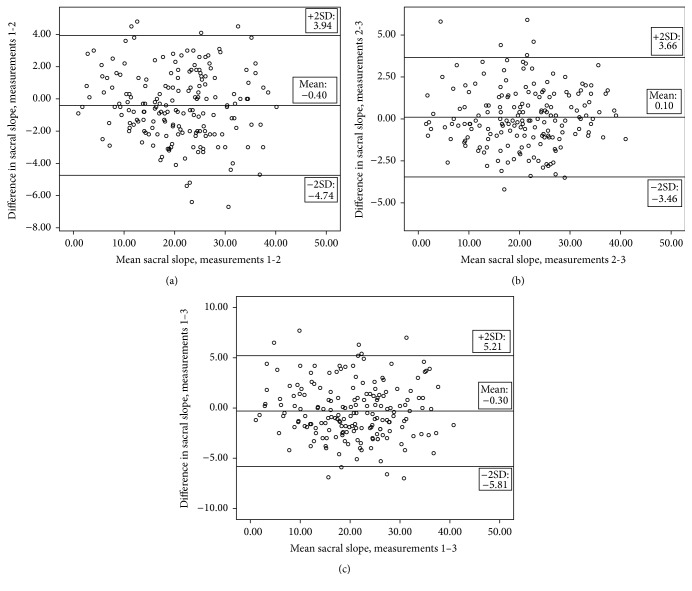
Bland and Altman plots for sacral slope measurements, indicating mean differences and 95% limits of agreement between (a) measurements 1 and 2, (b) measurements 2 and 3, and (c) measurements 1 and 3.

Agreement between the 3 testing occasions was presented through a graphical representation of mean differences and 95% LOAs (±2SDs of the differences) of all participants' values (Figures 2 and 3). These data acted complementarily to the descriptive data presented in Table 1 and the MDC values presented in Table 2(b).

### 3.4. Correlations between Sacral Slopes and Lumbar Curves

**Figure 4:**
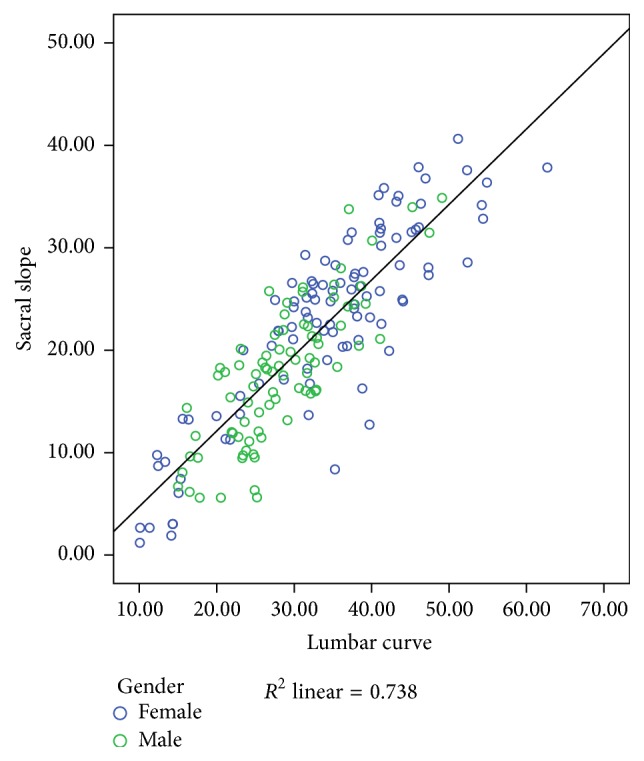
Scatterplot of the association between lumbar curve and sacral slope.

Pearson's correlation coefficient between the 2 angles was very high (*r* = 0.86, *p* < 0.001) (Figure 4). Similarly high correlations were found for each gender separately, *r* = 0.86 (*p* < 0.001) for females and *r* = 0.81 (*p* < 0.001) for males.

### 3.5. Differences in Sacral Slopes and Lumbar Curves between Male and Female Participants

**Figure 5:**
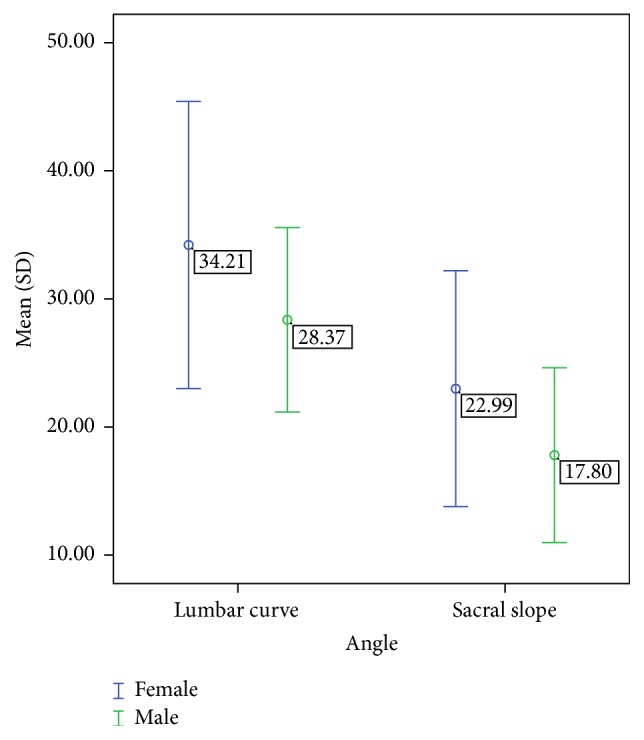
Between-gender differences (mean ± 1SD) separately for lumbar curve and sacral slope.

The method used was able to differentiate between male and female participants' data for both angle values measured. Between-gender means (SD) and mean differences are reported in Table 2(b) and a graphical representation is provided in Figure 5.

## 4. Discussion

One aim of our research was to examine the reliability of low back and pelvic posture with three sequential measurements on the same day and time of day, and another aim was to examine whether there is a correlation between lumbar curve and sacral slope and whether posture measurements differ between male and female participants. All subjects had no low back or pelvic pain for at least 2 years prior to the measurement, with the majority of the subjects never having a serious lumbopelvic pain incident in their life. Posture measurements were conducted with the use of the “iHandy Level” application for smartphones. The hypotheses of our research were that (a) low back curvature and pelvic tilt sagittal posture measurements could be measured with adequate intrarater reliability, which would be equally high for male and female participants, (b) sacral slope and lumbar curve angle data would be significantly associated, and (c) there would be significant between-gender standing low back and pelvic posture angle data, as measured by the method employed.

The study examined only spinal sagittal alignment, as it was easier to record with a common electronic goniometric smartphone application. Nevertheless, the risk factor of sustained loading positions in standing for low back pain development [10, 28] justifies the measurement of spinal and pelvic curves in the usual relaxed standing position of participants in our study. Among the methodological issues of our study contributing to the high reliability and agreement data obtained were that the 3 sequential measurements were conducted on the same day and time with the markings kept on participants' skin and the same rater measured each participant (intrarater reliability study). To control for raters blinding an independent third rater recorded all data. On the other hand, it can be argued that, by keeping the markings on participants, we may have improved the reliability of the test but moved away from clinical practice conditions. However, since a close relationship between inclinometric and smartphone measurements has already been shown [20] and due to the high between-day (1-week apart) intrarater reliability with removal of landmarks between measurements previously demonstrated for inclinometry [18], we believe that, with an accurate and consistent method of palpation of landmarks required, between-days reliability can also be achieved. This is a suggestion for future studies to validate.

High BMI was not an exclusion criterion in our study; however, it can make palpation of landmarks and sometimes placing of external measuring instruments difficult. Indeed, a very small percentage of our population (6/183 = 3.27%) had BMI over 30 kg/m^2^. Analytically, these were 3 females (with BMI's of 30.44, 30.48, and 31.24 kg/m^2^) and 3 males (with BMI's of 30.04, 30.61, and 32.85 kg/m^2^). This very small percentage of subjects with BMI > 30 kg/m^2^ is unlikely to have affected significantly the results of our overall population.

Our research examined test-retest, intrarater reliability with more than one raters. Each of the raters (2 involved) measured half of the subjects and no subject was measured by both of the raters involved. Our findings indicated that the reliability of “iHandy Level” application is high, based on the ICC, SEM, and MDC results (Table 2(b)) and agreement results (Figures 2 and 3). The same conclusion has also been reached in previous studies utilizing smartphones for goniometric measurements [19, 20]. Specifically Salam and Kolber (2014) reported an ICC of 0.81 for the lumbar curve angle measured in standing with an MDC of 7° at the 90% level, whereas in our study an ICC of 0.96 and of 0.97 for lumbar curve and sacral slope, respectively, was reported. The MDC_95%  CI_ in our study was 5.9° for the lumbar curvature and 4.46° for the pelvic tilt. Comparatively, the MDC_95%  CI_ for the lumbar curve from the Salamh and Kolber study was calculated at 8.31°, with our result being more accurate probably due to the much larger size of the population of our study. Bland and Altman plots show that there was less than 0.5° of a mean difference between all measurement occasions for both angles. Limits of agreement (±2SDs of the mean differences) magnitudes concur with the MDC_95%  CI_ data. An equal distribution of mean difference individual values around the mean overall difference was noted for both the lumbar curve (Figure 2) and sacral slope (Figure 3) data. We have observed very small improvement between measurements 2 and 3 agreement for both angles, compared to the agreement of measurement 1 with 2 and 3; however, this was not of clinical significance, within the 0.5° range. There was a trend for some of the repeated measurements to exceed 10° of difference. This was probably due to the variability in posture of some of the participants, and differences of such magnitude were previously noted [20] using the same smartphone application. It is, therefore, concluded that the “iHandy Level” application is a useful tool in the examination of the spine range with high reliability scores.

The correlation between the lumbar curve and sacral slope in the sagittal plane was high (Pearson's *r* = 0.86) and above 0.8 when examined for each gender separately in our study. Our results concur with previous research, which had similar findings in both healthy populations [29] and populations with spinal pathologies [30]. This is expected, as the total amount of lumbar lordosis is determined by the relationship of the superior endplate of S1 with the horizontal [30] and also as L5 contributes around 40% to the overall lumbar curve [22], with the lower endplate of L5 being in direct ligamentous connection with the sacrum. Indeed, the lumbopelvic complex has been extensively studied and has been found to work in parallel in order to accommodate the stability of this anatomical area [31]. However, the opposite finding of no correlation between those 2 variables has also been reported for asymptomatic adults [16, 32] and this disparity can most likely be explained by differences in the measuring instruments employed as well as differences between the populations examined. Also, the symmetry in the shape of the lumbar curve [17] or whether the lower segments that are associated with pelvic orientation contribute more to the lumbar curve may be pivotal to this association.

We have additionally demonstrated the ability of measuring spinopelvic alignment differences in standing between male and female participants. Several studies have confirmed there are between-gender differences in spinal posture [6, 33, 34]. There have also been links demonstrating between-gender differences in posture, that lead to loading differences between males and females [35]. Such loading differences between genders may be contributing in combination with possible other risk factors to back pain development [9]. Therefore, a measurement method needs to be at least sensitive to between-gender differences, apart from being accurate on a test-retest occasion, in order to be used clinically.

Therefore, both of the hypotheses of our research have been confirmed, although there are two limitations. The first is based on the limited age range of our participants (mean age 26), because the majority of them were college students, not to be able to generalize the results in younger or older populations. Secondly, future research could enhance the measurements of standing posture with habitual sitting postures, include low back and/or pelvic pain populations, and examine how their data differ from those of their healthy counterparts, in order to examine the effect of back pain on spinal posture reliability measurements. Finally, it has to be stated that the measurement method used does not take into account the shape of the lumbar curve and therefore a uniform curve is assumed for all participants. Differences in the shape of the curve as well as differences in intersegmental movement differences could potentially be important for spinal pathology and its evaluation.

## 5. Conclusions

The intrarater reliability of sagittal low back and pelvic alignment in standing on the same day and time of day has been confirmed and the construct validity of the spinal posture measurements was established through intercorrelations of both angles measured and between-gender comparison of relaxed upright lumbar and pelvic posture in standing.

## Figures and Tables

**Table 1:** Lumbar curve and sacral slope mean (SD) values for the 3 measurements, the mean of 3 measurements, and repeated measures ANOVA results.

Measurement	Lumbar curve°		Sacral slope°	
	Mean (SD)	ANOVA	Mean (SD)	ANOVA
1	31.29 (10.00)	*F*_(2,182)_ = 2.4, *p* = 0.092 > 0.05	20.40 (8.55)	*F*_(2,182)_ = 3.1, *p* = 0.047 ≈ 0.05
2	31.76 (10.30)		20.80 (8.77)	
3	31.63 (10.18)		20.70 (8.74)	
Mean	31.56 (10.01)		20.64 (8.59)	

**Table:** (a) Angle data in degrees [mean (SD)]. Lumbar curves and sacral slopes differed significantly between male and female participants

	Salamh and Kolber [20]	Current study			
Gender	Mixed (*n* = 30)	Male (*n* = 83)	Female (*n* = 100)	Total (*n* = 183)	Male-female mean difference

Lumbar curve°	32-33	28.37 (7.19)	34.21 (11.21)^*∗∗∗*^	31.56 (10.01)	5.84
Sacral slope°	—	17.80 (6.82)	22.99 (9.20)^*∗∗∗*^	20.64 (8.59)	5.18

^*∗∗∗*^ *p* < 0.001, significantly different to male values.

**Table:** (b) Intrarater reliability statistics

	Salamh and Kolber [20]			Current study								
Gender	Mixed (*n* = 30)			Male (*n* = 83)			Female (*n* = 100)			Total (*n* = 183)		

	ICC_3,*k*_ (95% CI)	SEM°	MDC_90% CI_°	ICC_2,1_ (95% CI)	SEM°	MDC_95% CI_°	ICC_2,1_ (95% CI)	SEM°	MDC_95% CI_°	ICC_2,1_ (95% CI)	SEM°	MDC_95% CI_°

Lumbar curve	0.81 (0.61–0.91)	3	7	0.93 (0.89–0.95)	2.02	5.59	0.96 (0.95–0.97)	2.23	6.17	0.96 (0.94–0.97)	2.13	5.9
Sacral slope	—	—	—	0.95 (0.93–0.96)	1.57	4.35	0.97 (0.96–0.98)	1.63	4.51	0.97 (0.96–0.97)	1.61	4.46

ICC: Intraclass Correlation Coefficient; SEM: Standard Error of the Measurement; MDC: Minimum Detectable Change.
